# Haematological parameters and biochemical indices of African catfish (*Clarias gariepinus*) exposed to glyphosate-based herbicide (Force up^®^) for 96 hours

**DOI:** 10.3389/ftox.2024.1448861

**Published:** 2024-11-12

**Authors:** Selim Adewale Alarape, Deborah Damilola Adeoye, Azeezat Oluwakemi Amusa, Olanike Kudirat Adeyemo

**Affiliations:** ^1^ Department of Veterinary Public Health and Preventive Medicine, Faculty of Veterinary Medicine, University of Ibadan, Ibadan, Nigeria; ^2^ Federal College of Animal Health and Production Technology, Moor Plantation, Ibadan, Nigeria

**Keywords:** herbicide, glyphosate, catfish, blood parameters, biochemical indices

## Abstract

**Introduction:**

Geometric aquaculture growth has resulted in exponentially increasing use of agrochemicals as either parasiticides or herbicides in the aquaculture environment. This study determines some of the toxicological (haematological and biochemical) effects of glyphosate-based herbicides on non-target aquatic animals using *Clarias gariepinus* as the animal model.

**Method:**

Seventy-five apparently healthy adult *C. gariepinus* (300 g) were sourced from a local farmer and acclimatised for 2 weeks; of these, sixty subjects were divided into four treatment groups (fifteen fish per group and five replicates per unit) by simple randomisation and labelled as T0 (control), T1 (first treatment), T2 (second treatment), and T3 (third treatment). The treatments were replicated thrice. Four concentrations of Force up^®^ [0 mL, 0.15 mL (0.003 mL/L or 5.1 mg/L), 0.225 mL (0.0045 mL/L or 7.65 mg/L), and 0.3 mL (0.006 mL/L or 10.2 mg/L) were added to a 50-L tank of water for T0, T1, T2, and T3, respectively. Approximately 5 mL of blood was collected from the fish in each treatment group 96 h post-exposure for measurement of the blood parameters and biochemical indices using standard analytical methods as well as calculation of the mean values.

**Result:**

The mean values of the packed cell volume, haemoglobin concentration, red blood cell count, and white blood cell count compared to the control group showed an initial increase at T1 but decreased as the glyphosate concentrations increased at T2 (0.0045 mL/L) and T3 (0.006 mL/L). The platelet mean values decreased at T1, increased at T2, and decreased at T3, while the mean values of the corpuscular volume, corpuscular haemoglobin, and corpuscular haemoglobin concentration increased with glyphosate concentration, with the mean corpuscular haemoglobin concentration decreasing at T2. Only the platelet value was statistically significant at a *p*-value of <0.05 using ANOVA and *post hoc* Tukey test. The biochemical indices showed decreases in the mean values of aspartate transaminase, blood urea nitrogen, creatinine, and triglycerides at T1, increases at T2, and decreases at T3, while the total protein (g/dL), cholesterol, alanine transaminase, and alkaline phosphatase values showed increases at T1 and decreases at T2 and T3. All these values were not statistically significant based on ANOVA and had *p*-values >0.05.

**Discussion:**

Based on the results of this study, it is deduced that glyphosate-based herbicide (Force up^®^) has genotoxic, hepatotoxic, and nephrotoxic effects on *C. gariepinus* even at sublethal doses, with more adverse effects at increasing concentrations.

## 1 Introduction

Global aquaculture production has quadrupled over the past 20 years and is likely to double over the next 15 years as a result of wild fisheries approaching their biological limits and the increasing global demand for cultured fish (Ayinla, 2012). The contamination of freshwater by different pollutants has posed serious global challenges in recent times owing to the undesirable effects on aquatic organisms (Muhammad et al., 2021). The flow of agrowastes such as herbicides in the form of runoffs from farmlands into streams, rivers, and lakes is a global concern that needs attention; this is because the exposure of fish and other aquatic biota to such chemicals can cause grievous physiological impairments as well as death to these organisms (Nikinmma, 2014). Humans rely on fish as a veritable source of protein food (Awoke et al., 2023); given the rise in fish farming and aquaculture, there has also been an exponential increase in the use of agrochemicals.

Agrochemicals refer to pesticides like insecticides, herbicides, and fungicides. These agrochemicals are often marketed by the manufacturers as harmless with little or no side effects. However, some researchers view agrochemicals as substances whose environmental effects are poorly understood despite their rapidly increasing use (Alarape et al., 2023). The constant flow of agricultural effluents into water bodies often leads to accumulation of various pollutants that becomes apparent when considering toxic pollution (Mason, 1991). The indiscriminate use of herbicides along with their careless handling, accidental spillage, or discharge of treated effluents into natural waterways can have harmful effects on fish and other forms of aquatic life, contributing to long-term effects on the environment (Akhtar et al., 1986). Herbicides are widely used to control the proliferation of water plants that impede the flow of water during summer, when sudden heavy rains can cause flooding (Annune et al., 1994). Although the direct effect of herbicide application is the loss of macrophytes, non-target organisms such as fish may also be affected through loss of habitat and food supply (Ervnest, 2004).

Water-soluble toxicants from agricultural runoff are rapidly finding their way into natural water bodies, where they ultimately decompose, volatilise, or sometimes form insoluble salts that precipitate and are incorporated into the sediment (Ezemonye and Tongo, 2009). Indiscriminate use of agrochemicals like herbicides by farmers to control weed growth in farms has resulted in harmful consequences to non-target organisms (FAO/WHO, 2016). Glyphosate is a non-target organophosphate herbicide whose solubility and mobility in hydrophilic solvents enable rapid leaching into the soil and subsequent contamination of the ground and surface water bodies, resulting in possible build-up in the aquatic food chain (Alarape et al., 2023). Glyphosate [N-(phosphonomethyl) glycine] is the most widely used herbicide globally (Duke and Powles, 2008); its popularity revolves around not only its efficiency in killing weeds at low cost but also its perceived low toxicity, rapid absorption by plants, and slow evolution of glyphosate resistance in weeds (Duke and Powles, 2008).

The US Environmental Protection Agency (EPA) has classified glyphosate as practically non-toxic and not an irritant under the acute toxicity classification system. This classification is primarily based on the toxicity data and its unique mode of action via a biochemical pathway that exists only in a small number of organisms (most of which are green plants) utilising the shikimic acid pathway to produce amino acids. This classification is supported by the majority of scientific literature on the toxic effects of glyphosate. However, in 2012, the Food and Agriculture Organization (FAO, 2012) reported that glyphosate and its major metabolite, amino methyl phosphoric acid (AMPA), are of toxicological concern mainly from accumulation of residues in the food chain. The FAO further states that the dietary risks of glyphosate and AMPA are unlikely if the maximum daily intake of 1 mg/kg bodyweight (bw) is not exceeded.

Even though there are reports (World Health Organization, 1994; Zouaoui et al., 2013) that pure glyphosate is non-toxic to human beings, glyphosate application in the agricultural sector involves mixing with surfactants such as polyoxyethykeneamine (POEA) and alkylpolyphosphate amine. Giesy et al. (2000) reported that the glyphosate-based herbicide Roundup^®^ can penetrate cell membranes, especially the gill cells of fish and other aquatic organisms, because it has POEA as the surfactant. Portier et al. (2016) and Portier (2020) reported that glyphosate formulations are probable human carcinogens.

AMPA is a primary product of glyphosate degradation along with non-toxic products like sarcosine and glycine. Unlike AMPA, which is 3–6 times more toxic and persistent than glyphosate (Sun et al., 2019), sarcosine is barely detected in the natural environment (Wang et al., 2016) except under experimental laboratory conditions (Zhan et al., 2018). AMPA belongs to the aminomethylenephosphonate chemical group and has a significant measured concentration in the environment (Tresnakova et al., 2021). Owing to its phosphonate and amine functional groups, AMPA forms metal complexes with Ca^2+^, Mg^2+^, Mn^2+^, and Zn^2+^ and is adsorbed firmly on soil (Poppov et al., 2001).

African catfish (*Clarias gariepinus*) is a significant commercial fish and is one of the common and widely consumed freshwater fish in Nigeria (Olaifa et al., 2003). This species is hardy and widespread in Nigeria’s natural and manmade water bodies (Awoke et al., 2023), which makes it a very good bioindicator for studying the responses of various pesticides. *C. gariepinus* exhibits structural and physiological changes in the presence of xenobiotics; this fish is highly cosmopolitan and is found in not only African rivers but also temporary puddles in arid areas after a bit of rain (Akeredolu et al., 2022).

Haematological analyses are commonly used to evaluate the health and welfare of fish under aquaculture conditions as well as in scientific studies to assess the influences of environmental factors (Fazio, 2019; Witeska et al., 2022). Blood indices are sensitive and fast-reacting biomarkers of various environmental impacts, including water pollution by toxic agents (Witeska et al., 2023). Blood parameters reflect a wide range of physiological conditions, both adaptive and disruptive, and provide extensive information on various physiological functions as reliable biomarkers of an organism’s performance. Blood sampling is less invasive compared to the collection of other tissues from live organisms and is possible under both laboratory and field conditions (Witeska et al., 2023). The basic haematological parameters include haematocrit (Ht), haemoglobin concentration (Hb), erythrocyte or red blood cell (RBC) count, and leucocyte or white blood cell (WBC) count, and blood smears can be obtained with only a small amount of blood. Additionally, some derived red blood parameters can also be calculated using Ht, Hb, RBC values, and appropriate formulae, such as the mean corpuscular volume (MCV), mean corpuscular haemoglobin (MCH), and mean corpuscular haemoglobin concentration (MCHC). Stained blood smears can be used for quantitative evaluation of the erythrocyte and leucocyte populations to calculate the percentage of immature erythrocytes (erythroblasts), erythrocyte cellular and nuclear anomalies, differential leucocyte count (DLC, which provides the proportions of various types of leukocytes), and thrombocyte count (TC). These parameters are useful for evaluating erythropoietic activity, cytotoxic and genotoxic effects, and the status of the immune system (Witeska et al., 2023).

Toxic agents like metal ions, pesticides, or other anthropogenic aquatic pollutants as well as pharmaceuticals such as immunomodulators, antimicrobial and antiparasitic therapeutics, or anaesthetics, have been proven to cause haematological changes in fish (Dias et al., 2023; Kanu et al., 2023; Rohani, 2023; Duman and Sahan, 2023; Moradi et al., 2022; Bentes et al., 2022; Kubra, 2022). Bojarski et al. (2022) demonstrated that haematological indices were the most sensitive and reliable biomarkers of exposure of *Cyprinus carpio* to the herbicide Roundup; in their study, fewer changes in the blood biochemical parameters were observed compared to the haematological ones, while the microstructures of the analysed organs (gills, liver, trunk, and kidney) were unchanged.

Haematological indices provide information on the oxygen transport capacity, immune status, stress response, cytotoxicity, and genotoxicity (Witeska et al., 2023). WBC count and leukogram or DLC are the most commonly used indicators of fish immune potential. Toxic agents often affect the leucocyte count and increases or decreases are observed similar to the case of the red blood parameters (Witeska et al., 2023). The TC is usually obtained indirectly by counting the thrombocytes in a smear and estimating the proportions of leukocytes and erythrocytes (Witeska et al., 2023). The TC in fish is highly variable (Witeska et al., 2016), and the thrombocytes are sometimes included in the leucocyte population as they are involved in both blood coagulation and defence mechanisms (Tavares-Dias and Oliveira, 2009; Stosik et al., 2019). Blood biochemical parameters can also be used to detect the health of fish (De Pedro et al., 2005). Exogenous factors, such as management (Svobodova et al., 2008), diseases (Chen et al., 2005), and stress (Cnaani et al., 2004), always induce major changes in the blood composition. Basic ecological factors, such as feeding regime and stocking density, also have direct influences on certain biochemical parameters (Coz-Rakovac et al., 2005).

Glyphosate concentrations over a wide range have been found to be poisonous to young catfish species, leading to poor growth, low survival, and death (FAO/WHO, 2016). Numerous surfactants have been detected in glyphosate formulations, which are noxious and hence not suitable for aquatic use (Tu et al., 2001).

Akeredolu et al. (2022) conducted a study to evaluate the acute toxicity as well as histological and genotoxic effects (erythrocytic nuclear abnormalities) of lethal and sublethal concentrations of three commonly used pesticides (atrazine, butachlor, and glyphosate) on *C. gariepinus*. The fish were exposed to the pesticides for 96-h periods to determine their LC_50_ and sublethal effects at various concentrations (1/10th, 1/100th, 1/1000th of 96-h LC_50_) over 28 days. The 96-h LC_50_ values were 7.63, 0.7, and 15.97 mg/L for atrazine, butachlor, and glyphosate, respectively.

Application of environmental toxicology studies to non-mammalian vertebrates is a rapidly expanding field for evaluating the effects of noxious compounds (Ayoola, 2008). Despite the identified harmful physiological effects of exposure to glyphosate, especially at high concentrations, there are limited attempts to evaluate the impacts of its low concentrations in water. Therefore, it is important to determine some of the toxicological (haematological and biochemical) effects of low concentrations of glyphosate-based herbicides present in water on the aquatic environment and animals using cultured *C. gariepinus* as the animal model.

## 2 Materials and methods

The trademarked chemical Force up^®^{glyphosate [N-(phosphonomethyl) glycine] solution} was obtained from a commercial vendor in Ibadan, Nigeria. The other requirements for the experiments were *C. gariepinus*, plastic keg, holding tanks, pelletised fish feed, distilled water, WBC diluting fluid, syringes and needles, ethylene diamine tetraacetate (EDTA) sample bottles, gloves, air pump/aerator machine, haemocytometer, glass slides, cover slips, capillary tubes, microscope, Bijou bottles, capillary pipette, Wintrobe haematocrit tubes, centrifuge, Drabkin’s solution, colorimeter, haemoglobinometer, spectrophotometer, H1 83200 multiparameter bench photometer, and H1 98186 dissolved oxygen meter.

### 2.1 Methods

Seventy-five apparently healthy adult *C. gariepinus* (Chordata, Osteichthyes, Siluriformes, Clariidae) weighing approximately 300 g each were purchased from a reputable farm in Ibadan, Nigeria. They were transported in a plastic keg to the Fish and Wildlife Laboratory at the Department of Veterinary Public Health and Preventive Medicine of the University of Ibadan, Nigeria, on the same day. The fish were subsequently transferred into holding tanks for 2 weeks to acclimatisation (temperature: 28.4°C ± 2°C; relative humidity: 78% ± 5%). During this period, the fish were fed protein pellets daily at 5% bw. The food remains were syphoned out of the aquaria daily during water exchange. The water was changed every 24 h and the fish were fed simultaneously; during the change, the aquaria were washed with a sponge and water, following which fresh water was replaced up to the 50 L mark. The water temperature, pH, and dissolved oxygen parameters were monitored.

After acclimatisation, sixty fish were divided into four groups by simple randomisation. The containers were labelled T0a, T0b, and T0c (controls); T1a, T1b, and T1c (first treatment); T2a, T2b, and T2c (second treatment); and T3a, T3b, and T3c (third treatment). Altogether, there were four treatment groups with fifteen fish per group and three replicas of five fish per treatment unit for each concentration. The fish were exposed to four different concentrations of the herbicide Force up^®^: 0.15 mL (0.003 mL/L or 5.10 mg/L) in T1, 0.225 mL (0.0045 mL/L or 7.65 mg/L) in T2, 0.3 mL (0.006 mL/L or 10.20 mg/L) in T3, and 0 mL as the control in T0. The chemicals at these concentrations were added to the 50-L tanks of water.

After adding the chemical, the water was aerated for approximately 1 h daily while the physical observations and behavioural changes were noted after introducing Force up^®^. Fresh solutions of the herbicide were prepared every 24 h and the aeration was performed daily for 5 days.

#### 2.2 Biochemical and haematological analyses

Blood samples were collected from each treatment group for haematological and biochemical analyses at the beginning and end of the experiment. Blood was drawn from the caudal peduncle of the fish using a disposable needle and syringe into an EDTA sample bottle to avoid clotting. The packed cell volume (PCV) was analysed with a microhaematocrit via heparinised 25-mm capillary tubes. The RBC and WBC counts were determined as described by Blaxhall and Daisley (1973), while Hb was estimated using the method outlined by Wedemeyer and Yasutake (1977). The other haematological indices like the MCH, MCV, and MCHC were determined using the following formulae reported by Dacie and Lewis (1984):

$$\text{MCH }\left(\text{pg}\right)=\left[\text{Hb }\left({g\;\text{dL}}^{-1}\right)\times 10\right]/\text{ RBC }\left(10^{6}\;\mu L^{-1}\right),$$

$$\text{MCV }\left(\text{fL}\right)=\left(\text{Ht}\times 10\right)/\text{RBC }\left(10^{6}\;\mu L^{-1}\right),$$

$$\text{MCHC }\left(\%\right)=\left[\text{Hb }\left({g\;\text{dL}}^{-1}\right)\times 100\right]/\text{Ht}.$$

Biochemical estimates of the blood creatinine, blood protein, blood cholesterol, aspartate transaminase (AST), alanine transaminase (ALT), alkaline phosphatase (ALP), triglycerides, and blood urea nitrogen (BUN) were determined using standard methods (Annino, 1976; Henry, 1968; Fawcette and Scott, 1960; Folin and Wu, 1920).

### 2.3 Data analysis

The obtained data were analysed using SPSS Statistics Version 26, and the results were expressed as mean ± standard deviation (SD). The average values were compared using one-way analysis of variance (ANOVA) and considerable variations among the treatment groups were determined by the *post hoc* Tukey test using SPSS for Windows. The degree of significance was set at *p* <0.05.

### 2.4 Ethical consideration

The Animal Care and Use in Research Committee of the University of Ibadan accepted and approved the research protocols in this study under the number UI-ACUREC/019–0220/6.

## 3 Results

### 3.1 Haematological parameters

As seen in Table 1, the mean PCV, Hb, RBC count, and WBC count showed initial increases (in T1) for the lowest glyphosate herbicide concentration used in this study (0.003 mL/L) compared to the control group; this trend reduced as the glyphosate concentrations increased in T2 (0.0045 mL/L) and T3 (0.006 mL/L). The platelet value decreased for the lowest glyphosate concentration in T1, increased in T2, and was lowest in T3. Meanwhile, the MCV and MCH values both increased in T1, T2, and T3 compared to the mean value in the control group, with the highest values noted in T3; the MCHC increased in T1, reduced in T2, and increased in T3. Table 2 shows the results of one-way ANOVA for the haematological parameters along with their respective F and *p* values. Only the platelet *p*-value of 0.049 was statistically significant in the Tukey honestly significant difference (HSD) *post hoc* test (*p* <0.05) in T2 *versus* T3, as shown in Table 3.

**TABLE 1:** Values of the mean ± standard deviation (SD) of the haematological parameters of *Clarias gariepinus* exposed to glyphosate herbicide (Force up®) for 96 h at different treatment concentrations.

Parameter	Treatment group	Mean ± SD
PCV (%)	1	30.00 ± 5.29
	2	34.67 ± 9.71
	3	24.33 ± 3.52
	4	21.00 ± 1.73
Hb (g/dL)	1	9.53 ± 1.75
	2	11.33 ± 3.25
	3	7.67 ± 0.90
	4	6.70 ± 0.26
RBC (×10^6^ mm^3^)	1	2.52 ± 0.93
	2	2.93 ± 1.12
	3	2.10 ± 1.07
	4	1.33 ± 0.08
WBC (mm^3^)	1	15,167.00 ± 975.11
	2	16,283.00 ± 3,755.77
	3	14,017.00 ± 1,300.32
	4	13,367.00 ± 678.85
Platelets (mm^3^)	1	138,000.00 ± 4,000.00
	2	118,330.00 ± 7,637.63
	3	150,000.00 ± 15,099.67
	4	79,333.00 ± 49,903.24
MCV (fL)	1	112.56 ± 30.13
	2	123.18 ± 19.61
	3	129.43 ± 39.34
	4	157.37 ± 4.65
MCHC (%)	1	31.76 ± 0.56
	2	32.65 ± 0.32
	3	31.59 ± 0.84
	4	31.64 ± 1.08
MCH (pg)	1	35.74 ± 9.52
	2	40.21 ± 6.29
	3	41.09 ± 13.32
	4	50.30 ± 1.46

^a^ 1 = T0 (Control); 2 = T1 (0.003 mL/L); 3 = T2 (0.045 mL/L); 4 = T3 (0.006 mL/L).

**TABLE 2:** F-values and *p*-values of the haematological parameters of *C. gariepinus* exposed to glyphosate herbicide (Force up^®^) for 96 h at different treatment concentrations.

Parameter		Sum of squares	df	Mean square	F-value	*p*-value (sig.)
PCV (%)	Between groups	329.67	3	109.89	3.19	0.08
	Within groups	275.33	8	34.42		
	Total	605.00	11			
Hb (g/dL)	Between groups	37.95	3	12.65	3.49	0.07
	Within groups	29.02	8	3.63		
	Total	66.97	11			
RBC (×10^6^ mm^3^)	Between groups	4.17	3	1.39	1.71	0.24
	Within groups	6.51	8	0.81		
	Total	10.68	11			
WBC (mm^3^)	Between groups	1.49×10^7^	3	4,969,166.67	1.16	0.39
	Within groups	3.44 ×10^7^	8	4,302,083.33		
	Total	4.93 ×10^7^	11			
Platelets (mm^3^)	Between groups	8.618×10^9^	3	2.87×10^9^	4.11	0.05
	Within groups	5.59 ×10^9^	8	6.98 ×10^8^		
	Total	1.42 ×10^10^	11			
MCV (fL)	Between groups	3,295.63	3	1,098.55	1.54	0.28
	Within groups	5,723.33	8	715.42		
	Total	9,018.967	11			
MCHC (%)	Between groups	2.25	3	0.75	1.32	0.33
	Within groups	4.55	8	0.57		
	Total	6.80	11			
MCH (pg)	Between groups	335.82	3	111.94	1.45	0.30
	Within groups	619.50	8	77.44		
	Total	955.31	11			

*p*value ≤0.05 is statistically significant; df, degree of freedom.

**TABLE 3:** *Post hoc* (Tukey) values of the haematological parameters of *C. gariepinus* exposed to glyphosate herbicide (Force up^®^) for 96 h at different treatment concentrations.

Dependent variable (parameter)	Treatment group versus	Treatment group	*p*-value (sig.)
PCV (%)	1	2	0.77
		3	0.65
		4	0.31
	2	1	0.77
		3	0.22
		4	0.08
	3	1	0.65
		2	0.22
		4	0.90
	4	1	0.31
		2	0.08
		3	0.90
Hb (g/dL)	1	2	0.67
		3	0.64
		4	0.33
	2	1	0.67
		3	0.16
		4	0.07
	3	1	0.64
		2	0.16
		4	0.92
	4	1	0.33
		2	0.07
		3	0.92
RBC (×10^6^ mm^3^)	1	2	0.94
		3	0.94
		4	0.43
	2	1	0.94
		3	0.69
		4	0.21
	3	1	0.94
		2	0.69
		4	0.73
	4	1	0.43
		2	0.21
		3	0.73
WBC (mm^3^)	1	2	0.91
		3	0.90
		4	0.72
	2	1	0.91
		3	0.57
		4	0.37
	3	1	0.90
		2	0.57
		4	0.98
	4	1	0.72
		2	0.37
		3	0.98
Platelets (mm^3^)	1	2	0.80
		3	0.94
		4	0.10
	2	1	0.80
		3	0.50
		4	0.34
	3	1	0.94
		2	0.50
		4	0.05
	4	1	0.10
		2	0.34
		3	0.05
MCV (fL)	1	2	0.96
		3	0.87
		4	0.25
	2	1	0.96
		3	0.99
		4	0.45
	3	1	0.87
		2	0.99
		4	0.60
	4	1	0.25
		2	0.45
		3	0.60
MCHC (%)	1	2	0.51
		3	1.00
		4	1.00
	2	1	0.51
		3	0.37
		4	0.41
	3	1	0.99
		2	0.37
		4	1.00
	4	1	1.00
		2	0.41
		3	1.00
MCH (pg)	1	2	0.92
		3	0.88
		4	0.26
	2	1	0.92
		3	1.00
		4	0.53
	3	1	0.88
		2	1.00
		4	0.60
	4	1	0.26
		2	0.53
		3	0.60

^a^ The mean difference is significant at the 0.05 level.

^b^ 1 = T0 (Control); 2 = T1 (0.003 mL/L); 3 = T2 (0.045 mL/L); 4 = T3 (0.006 mL/L).

### 3.2 Biochemical indices

The biochemical indices listed in Table 4 showed decreases in the mean values of AST, BUN, creatinine, and triglycerides for T1, with corresponding increases in the values for T2 and lowest values in T3 compared to the control group; however, the total protein, ALT, ALP, and cholesterol values initially increased in T1 but decreased thereafter in T2 and T3. The values of all biochemical indices were not statistically significant, with no differences seen between the treatment groups based on ANOVA and Tukey HSD *post hoc* test, as shown in Tables 5, 6.

**TABLE 4:** Values of mean ± SD of the biochemical indices of *C. gariepinus* exposed to glyphosate herbicide (Force up®) for 96 h at different treatment concentrations.

Parameter	Treatment group	Mean ± SD
Total protein (g/dL)	1	5.83 ± 0.35
	2	5.87 ± 0.90
	3	5.70 ± 0.53
	4	4.83 ± 0.29
AST (μL)	1	293.33 ± 11.55
	2	276.67 ± 68.72
	3	300.33 ± 34.65
	4	226.67 ± 41.30
ALT (μL)	1	37.00 ± 5.57
	2	37.67 ± 9.29
	3	36.33 ± 7.77
	4	25.33 ± 4.73
ALP (μL)	1	516.00 ± 55.68
	2	522.00 ± 52.57
	3	480.67 ± 91.44
	4	376.00 ± 79.73
BUN (mg/dL)	1	7.87 ± 0.64
	2	7.50 ± 0.17
	3	7.73 ± 0.70
	4	7.07 ± 0.76
Creatinine (mg/dL)	1	0.63 ± 0.06
	2	0.60 ± 0.00
	3	0.63 ± 0.06
	4	0.57 ± 0.06
Cholesterol (mg/dL)	1	195.33 ± 4.51
	2	198.67 ± 19.01
	3	196.33 ± 12.66
	4	182.33 ± 5.13
Triglycerides (mg/dL)	1	63.67 ± 1.53
	2	57.33 ± 12.70
	3	64.00 ± 4.00
	4	51.33 ± 3.51

^a^ 1 = T0 (Control); 2 = T1 (0.003 mL/L); 3 = T2 (0.045 mL/L); 4 = T3 (0.006 mL/L).

**TABLE 5:** F-values and *p*-values of the biochemical indices of *C. gariepinus* exposed to glyphosate herbicide (Force up^®^) for 96 h at different treatment concentrations.

Parameter		Sum of squares	df	Mean square	F-value	*p*-value (Sig.)
Total protein (g/dL)	Between groups	2.15	3	0.72	2.20	0.17
	Within groups	2.60	8	0.33		
	Total	4.75	11			
AST (μL)	Between groups	9,943.58	3	3,314.53	1.71	0.24
	Within groups	15,522.67	8	1,940.33		
	Total	25,466.25	11			
ALT (μL)	Between groups	308.93	3	102.97	2.06	0.18
	Within groups	400.00	8	50.00		
	Total	708.92	11			
ALP (μL)	Between groups	41,148.00	3	13,716.00	2.67	0.12
	Within groups	41,164.67	8	5,145.58		
	Total	82,312.67	11			
BUN (mg/dL)	Between groups	1.11	3	0.37	0.99	0.45
	Within groups	3.00	8	0.38		
	Total	4.11	11			
Creatinine (mg/dL)	Between groups	0.01	3	0.00	1.22	0.36
	Within groups	0.02	8	0.00		
	Total	0.03	11			
Cholesterol (mg/dL)	Between groups	487.00	3	162.33	1.14	0.39
	Within groups	1,136.67	8	142.08		
	Total	1,623.67	11			
Triglycerides (mg/dL)	Between groups	324.92	3	108.31	2.26	0.16
	Within groups	384.00	8	48.00		
	Total	708.92	11			

^a^ value ≤0.05 is statistically significant; df, degree of freedom.

**TABLE 6:** *Post hoc* (Tukey) values of the haematological parameters of *C. gariepinus* exposed to glyphosate herbicide (Force up^®^) for 96 h at different treatment concentrations.

Dependent variable (parameter)	Treatment group versus	Treatment group	*p*-value (Sig.)
Total protein (g/dL)	1	2	1.00
		3	0.99
		4	0.22
	2	1	1.00
		3	0.98
		4	0.20
	3	1	0.99
		2	0.98
		4	0.32
	4	1	0.22
		2	0.20
		3	0.32
AST (μL)	1	2	0.97
		3	1.00
		4	0.32
	2	1	0.97
		3	0.91
		4	0.54
	3	1	1.00
		2	0.91
		4	0.25
	4	1	0.32
		2	0.54
		3	0.25
ALT (μL)	1	2	1.00
		3	1.00
		4	0.26
	2	1	1.00
		3	1.00
		4	0.22
	3	1	1.00
		2	1.00
		4	0.30
	4	1	0.26
		2	0.22
		3	0.30
ALP (μL)	1	2	1.00
		3	0.93
		4	0.16
	2	1	1.00
		3	0.89
		4	0.14
	3	1	0.93
		2	0.89
		4	0.35
	4	1	0.16
		2	0.14
		3	0.35
BUN (mg/dL)	1	2	0.88
		3	0.99
		4	0.43
	2	1	0.88
		3	0.96
		4	0.82
	3	1	0.99
		2	0.96
		4	0.57
	4	1	0.43
		2	0.82
		3	0.57
Creatinine (mg/dL)	1	2	0.85
		3	1.00
		4	0.41
	2	1	0.85
		3	0.85
		4	0.85
	3	1	1.00
		2	0.85
		4	0.41
	4	1	0.41
		2	0.85
		3	0.41
Cholesterol (mg/dL)	1	2	0.99
		3	1.00
		4	0.57
	2	1	0.99
		3	1.00
		4	0.39
	3	1	1.00
		2	1.00
		4	0.51
	4	1	0.57
		2	0.39
		3	0.51
Triglycerides (mg/dL)	1	2	0.69
		3	1.00
		4	0.21
	2	1	0.69
		3	0.66
		4	0.72
	3	1	1.00
		2	0.66
		4	0.19
	4	1	0.21
		2	0.72
		3	0.19

^a^ The mean difference is significant at the 0.05 level.

^b^ 1 = T0 (Control); 2 = T1 (0.003 mL/L); 3 = T2 (0.045 mL/L); 4 = T3 (0.006 mL/L).

## 4 Discussion

Haematological indices are very important in the diagnoses of fish health, especially under different strenuous situations (Ramesh et al., 2009). The constituents of blood are highly vulnerable to chemicals such that physiological variations often manifest in the standards of some blood characteristics (Nwani et al., 2012). The RBCs of fish are suitable biomarkers for monitoring the water quality in an aquatic environment (Popoola, 2018); they are also helpful in evaluating the damage to organs and the consequent physiological, biochemical, and behavioural disorders in non-target animals.

Blood is a good indicator of the pathological condition of an animal exposed to any toxic compound (Olafedehan et al., 2010). Haematological parameters are typically used to monitor and diagnose alterations in an animal’s body or investigate blood damage caused by toxicity or disease (Merck, 2012; Togun et al., 2007; Onyeyelli et al., 1991). The RBC parameters include Ht, Hb, erythrocyte count, MCV, MCH, and MCHC (Witeska et al., 2023). The observed toxicity-induced changes may differ and show increases or decreases in the values of all or some of these parameters, indicating changes in the oxygen transport capacity. Increases in Ht, Hb, RBC count, or MCV may occur as compensatory responses to facilitate oxygen transport under general stress caused by a toxic agent, impair gas exchange by affecting the gill epithelium, or activate fish metabolism by increasing the detoxification pathways. Decreases in these parameters are observed when a toxic agent causes damage to the circulating erythrocytes, causes direct haemolysis during circulation, shortens the erythrocyte lifespan, or/and impairs erythropoiesis. Such changes are often described as anaemic responses (Witeska et al., 2023).

As these changes were consistent with the observations in this study (Table 1), we can infer that the initial increases in the PCV, Hb, RBC count, and MCV may be compensatory responses to glyphosate toxicity, whereas the anaemic responses in T2 and T3 may be because of increased concentrations of glyphosate causing RBC damage as the decreases in these parameter values were time and dose dependent. The reductions in these haematological indices may be a result of haemolysis of the RBCs, haemodilution owing to weakened osmoregulation across the gill epithelial cells, or significant deterioration in haematopoiesis (Ezike et al., 2019).

The decreases in the blood parameters (PCV, Hb, and RBC count) seen in this study (Table 1) are similar to those reported by Jasper et al. (2012) and indicate anaemic syndrome. They are also directly related to the increasing concentrations of herbicide and time of exposure (Sanam et al., 2019). Jasper et al. (2012) additionally reported that lower erythrocyte count, lower Hb with reduced Ht, and higher MCV are characteristics of macrocytic anaemia similar to the observations in this study (Zorriehzwhra et al., 2010). Haider and Rauf (2014) reported significant decreases in the RBC count, Hb, and Ht compared to the controls in *Cirrhinus mrigala* after chronic exposure to the organophosphate insecticide diazinon; according to the authors, the anaemic responses are attributable to the failure or suppression of the haematopoietic system of the fish. Javed et al. (2016) also reported macrocytic hypochromic anaemia (considerable decreases in Ht, Hb, RBC count, and MCHC with increases in MCV and MCH) that caused strong impairment of the oxygen carrying capacity in *Channa punctatus* subjected to industrial effluents containing metal-ion mixtures of Co, Cr, Cu, Fe, Mn, Ni, and Zn; they interpreted the anaemia through the inhibition of erythropoiesis. These findings are all similar to the observations of the present study. According to Kwiatkowska et al. (2014), glyphosate and its metabolites cause haemolysis and haemoglobin oxidation, and the changes in the erythrocytes increase with concentration. These changes were attributable only to poisoning with chemicals and genotoxicity in the erythrocytes and gill cells of fish (Moreno et al., 2014).

The WBCs and platelets increased in T1 and T2 (Table 1) compared to the controls, similar to the findings reported by George and Shukla (2013), and were attributed to oxidative stress. An increase in WBCs or leucocytosis is usually interpreted as activation of immune responses due to tissue damage by a toxic agent and often accompanied by neutrophilia or/and monocytosis, indicating an inflammatory response. However, leukopenia or decrease in WBCs is attributed to toxicity-induced general stress responses that particularly cause lymphopenia and increase in neutrophil to lymphocyte ratio or specific toxic actions affecting the circulating leukocytes or leukopoiesis resulting in immunosuppression (Witeska et al., 2023). Thus, the initial leucocytosis and subsequent leukopenia observed in the present study indicate stress caused by the glyphosate concentration in the water and resultant effects on the WBCs of the fish.

Lymphopenia or decreased lymphocytes observed in this study is similar to that found in rats and attributed to poisonous strain on non-specific tissues, followed by production of free radicals and prostaglandins that cause inflammatory reactions through neutrophilia and lymphopenia (Khan et al., 2013). Ligina et al. (2022) reported leucocytosis in *Anabas testudineus* upon intoxication with acrylamide. According to Zahran et al. (2018), exposure to the insecticide chlorpyrifos caused leucocytosis in *Oreochromis niloticus*; the numbers of both neutrophils and lymphocytes increased, but the increase in neutrophil count (neutrophilia) was more pronounced. The authors interpreted these changes as compensatory actions to the potentially compromised immune functions.

Bujjamma and Padmavathi (2018) also reported a concentration-dependent increase in WBCs in *Heteropneustes fossilis* exposed to cadmium; according to the authors, this may have resulted from immunomodulation caused by cadmium-induced tissue damage. According to Javed et al. (2016), who observed increased WBCs in *C. punctatus* subjected to power plant effluent containing a mixture of metal ions, leucocytosis was related to the magnitudes of damage and stress induced by heavy metals, which may have stimulated their immunological defences. Leucocytosis caused by azithromycin was reported by Shiogiri et al. (2010) in *O. niloticus*. According to Mahboub et al. (2021), *O. niloticus* exposed to mercury developed leucocytosis, lymphopenia, and neutrophilia accompanied by impaired immune functions.

Oluah et al. (2020) observed leucocytosis, lymphocytosis, neutropenia, and monocytopenia in *C. gariepinus* exposed to the herbicide Ronstar.

Leukopenia was also reported in *Sebastes schlegelii* exposed to ammonia (Shin et al., 2016) due to induced stress, while Tavares-Dias et al. (2011) observed a decrease in WBCs (both lymphocyte and neutrophil counts) in *Colossoma macropomum* exposed to Cu. According to Witeska and Kosciuk (2003), *C. carpio* subjected to acute exposure to Zn showed stress-related thrombocytosis. Lemly (2002) observed thrombocytosis in *Lepomis cyanellus* from Belews Lake contaminated with Se. Corredor-Santamaria et al. (2016) reported thrombocytosis in *Astyanax bimaculatus* and *Aequidens metae* from Ocoa River polluted with domestic and industrial wastewater; the authors attributed these findings to pollution-related increase in the defence responses. Fredianelli et al. (2019) reported thrombocytopenia in *Rhamdia quelen* that were sublethally intoxicated with the pesticide fipronil and attributed it to stress-related cortisol secretion as well as its action in reducing the quantity and quality of thrombocytes. Khan et al. (2016) also reported different responses of thrombocytes to glyphosate and atrazine exposure; the former herbicide induced an increase in thrombocytes, while the latter caused a decline. Thrombocytopenia was also observed by Omoregie and Oyebani (2002) in *O. niloticus* after treatment with oxytetracyclines. All of the above findings are consistent with those in the present study.

The biochemical indices considered in this study include total protein, albumin, globulin, AST, ALT, serum ALP, BUN, creatinine, cholesterol, and triglycerides. Exogenous factors, such as management (Svobodova et al., 2008), diseases (Chen et al., 2005), and stress (Cnaani et al., 2004), are known to always induce major changes in blood composition. For example, significant fluctuations were detected in the concentrations of cortisol, glucose, cholesterol, and other basic components in response to handling and hypoxic stresses (Skjervold et al., 2001). The blood levels of cortisol and glucose are considered to be specific indicators of sympathetic activation during stress conditions (Lermen et al., 2004).

The initial increase in the plasma protein concentration noted in this study (Table 4) may be caused by structural liver alternations that reduce amino transaminase activity with concurrent reduction in the deamination capacity (Kavadias et al., 2004). Bano (1985) observed an increase in the serum cholesterol level after pesticide administration. A high blood urea concentration was observed in *M. cephalus* by Borges et al. (2007), which could likely be a sign of stress associated with increased cortisol level.

Biochemical markers of hepatic and renal function as well as oxidative stress are important for monitoring exposure to environmental pollutants like glyphosate (Ahmad et al., 2004). Many pollutants can induce damages in biological systems, including the liver, which is the main organ for detoxification and biotransformation processes (Ahmad et al., 2000; Harish and Murugan, 2011). Balint et al. (1997) reported that ALT and AST that metabolise amino acids deviate from their normal levels and are good indicators of hepatic toxicity. Sarhan and Al-Sahhaf (2011) reported increases in AST, ALT, BUN, and creatinine levels compared to the controls because of hepatic and renal toxicities.

Among the different types of liver enzymes, AST and ALT have been proven to play essential roles in the metabolism of proteins and carbohydrates; hence, they are reliable indicators of liver damage caused by pesticides (Gholami-Seyedkolaei et al., 2013). In the present study, the results show increases in ALT and ALP in the groups exposed to glyphosate compared to the control (Table 4). This indicates that the concentrations used in this study are hepatotoxic to fish. There are also increases in the mean values of creatinine and BUN in T2 (Table 4), indicating adverse effects on both the liver (increased values of total bilirubin and conjugated bilirubin) and kidneys (high creatinine).

## 5 Conclusion and recommendations

Haematological variables are sensitive and reliable indicators of environmental impacts on fish, including those from toxic agents. They may show either the destructive effects of toxicity, such as anaemia and immunosuppression, or compensatory effects, such as increase in blood oxygen transport capacity or inflammation. Often, the haematological changes in fish subjected to toxicity reflect general and non-specific stress responses with unclear mechanisms (Witeska et al., 2023).

Glyphosate is used in agriculture as a standalone compound or component of commercial products, and its main metabolite AMPA may have adverse effects on freshwater and marine organisms at different levels of biological organisation. Glyphosate mainly induces oxidative stress and affects the antioxidant enzymes as well as blood parameters to cause several histopathological changes in the gills, liver, and kidneys of fish, in addition to genotoxicity, immunotoxicity, and cardiotoxicity (Tresnakova et al., 2021).

Based on the results of this study, it is clear that glyphosate-based herbicides (e.g., Force up^®^) have genotoxic, hepatotoxic, and nephrotoxic effects on *C. gariepinus*, with more adverse effects with increases in the concentration and time of exposure. Fish exposed to acute concentrations of the herbicide developed haematological and biochemical modifications that are likely detrimental to their survival and general wellbeing.

Glyphosate-based herbicides are the most heavily used substances in agriculture globally. Their toxicity can also end up in humans through the food chain. Therefore, suitable measures are recommended for the controlled and regular use of herbicides. This will help in utilising the beneficial effects without polluting the environment or leaving residues in food and water sources that can ultimately have potential negative effects on human health. The use of glyphosate-based herbicides near aquatic environment should be discouraged; furthermore, when used, the levels should be monitored to ensure appropriate concentrations that will not have adverse effects on non-target organisms. Mechanical and biological methods should also be encouraged as alternatives to chemical methods for weed control.

Governments and other relevant regulatory bodies should enlighten farmers and the general public regarding the adverse effects of glyphosate-based herbicides on aquatic animals and non-target organisms as well as its residual effects on both human and environmental health.

We also recommend the establishment of environmental monitoring agencies to periodically assess the cautious use of glyphosate in the environment. This will help mitigate the ecotoxicological consequences associated with herbicide use and prevent the risk of contamination to humans.

## Data Availability

The raw data supporting the conclusions of this article will be made available by the authors, without undue reservation.

## Abbreviations

ALP, alkaline phosphatase; ALT, alanine transaminase; AMPA: amino methyl phosphoric acid; AST, aspartate transaminase; BUN, blood urea nitrogen; EDTA, ethylene diamine tetraacetate; Hb: haemoglobin concentration; Ht: haematocrit; MCH, mean corpuscular haemoglobin; MCHC, mean corpuscular haemoglobin concentration; MCV, mean corpuscular volume; PCV, packed cell volume; RBC, red blood cell; SD, standard deviation; WBC, white blood cell.
